# Force fluctuations regulation and the role of neurophysiological mechanisms throughout different isometric contraction intensities

**DOI:** 10.1038/s41598-025-14543-6

**Published:** 2025-08-04

**Authors:** João H. Oliveira, João S. Gomes, Philipp Bauer, Pedro Pezarat-Correia, João R. Vaz

**Affiliations:** 1 Neuromuscular Research Laboratory, Faculty of Human Kinetics, University of Lisbon, Lisbon, Portugal; 2 CIPER, Faculty of Human Kinetics, University of Lisbon, Lisbon, Portugal; 3 Egas Moniz Center for Interdisciplinary Research (CiiEM), Egas Moniz School of Health & Science, Caparica, Almada, Portugal; 4 Egas Moniz School of Health & Science, Campus Universitário, Quinta da Granja, Monte de Caparica, Caparica, 2829-511 Portugal

**Keywords:** Complexity, Variability, Entropy, Motor control, Force control, Neuromuscular coordination, Neurophysiology, Physiology

## Abstract

Force complexity is a key indicator of the neuromuscular system’s adaptability and motor control. Although an inverted U-shaped relationship between force complexity and contraction intensity is established, its underlying mechanisms remain unclear. To investigate whether changes in motor unit behaviour (recruitment and firing rate) would accompany and explain this relationship, 25 young male adults performed a 30-second knee extensors’ hold-isometric task at 50%, 75%, 100%, 150% and 175% of their End-Test Torque (ETT), at individual’s optimal angle. Force complexity and motor unit behaviour were assessed through Sample Entropy (SampEn) and high-density surface electromyography, respectively. We demonstrated a trend for an inverted U-shaped relationship between force complexity and contraction intensity, with SampEn at ETT and 150%ETT being significantly higher than at 50%ETT and 75%ETT (all *p* < 0.05). This pattern was accompanied by an increase in motor unit actions potentials and firing rate as the intensity increased up to 150%ETT (all *p* < 0.05). A multiple linear regression analysis showed that force complexity was explained in 18% by the *vastus lateralis*’ motor unit behaviour. The findings suggest that changes in force complexity depend on contraction intensity and are partly explained by alterations in motor unit behaviour, influencing the neuromuscular system’s adaptability to meet task demands.

## Introduction

Healthy physiological outputs are characterized by constant inherent fluctuations which reflect the interplay between the multiple components of the systems, and respective feedback loops that operate over multiple temporal and spatial scales^1,2^. The temporal structure, or complexity, of these fluctuations is thought to contain key information about the state and functionality of the system^1,3–5^ and is considered an indicator of the system´s adaptability, i.e., the capacity of the system to be more flexible and to adapt to environmental challenges^6,7^. Specifically, force complexity has been suggested to be a key marker of the ability of the neuromuscular system to explore and achieve motor solutions in order to adapt the motor output to the task demands, i.e. the adaptability of the neuromuscular system^5^. Therefore, force complexity reflects the interactions between components of the neuromuscular system, such as motor cortical neurons, spinal motoneurons, muscle fibres, and muscle afferents, providing information on how these components interact with each other to produce complex patterns of force with the aim of achieving the task goals^5,8–10^. In fact, force complexity is considered an important measure of motor control and has been proposed as an indicator of the neuromuscular system’s functionality^11,12^. Interestingly, force complexity is thought to be negatively influenced by the ageing process^5,13^ disease states^8^ and neuromuscular fatigue^14–16^.

Despite the growing scientific interest in the neurophysiological processes underlying force complexity and, therefore, its potential translation to clinical settings, there is still no consensus on an appropriate setup for adequate assessment of such motor control properties. Given the nature of the commonly used algorithms to extract the nonlinear features of the signals, a common approach in the study of force variability and complexity involves sustained isometric contractions set to a certain percentage of the maximal voluntary isometric contraction^17^. However, previous studies demonstrated that force complexity is intensity-dependent^18^. Specifically, an inverted U-shape relationship was observed, where the higher levels of complexity were observed at ~ 30–40% of the maximum voluntary isometric contraction (MVIC). More recently, Fiogbe and colleagues^13^ investigating whether force complexity would change with increasing contraction intensities up to 40% of MVIC, found a positive relationship between force complexity and contraction intensity for the knee extensors, consistent with the ascendent part of the inverted U-shape relationship curve. Moreover, recent findings strengthen the importance of the contraction intensity for an adequate assessment and interpretation of force control. For example, Pethick et al.^11^ have found that the loss of complexity associated to the neuromuscular fatigue only occurs above the critical torque (CT), i.e. the maximal isometric torque that can be maintained without fatigue development^19^.

Although the relationship between force complexity and contraction intensity is well documented in the literature, the physiological mechanisms which explain this relationship are yet to be determined. Slifkin and Newell^18^ have raised the hypothesis that the inverted U-shape relationship could be explained by the control strategies of motor unit behaviour. Specifically, the authors have suggested that the higher levels of force complexity observed at 30–40% MVIC could be attributed to the larger availability of motor unit recruitment and firing rate strategies at this intensity range, providing the neuromuscular system greater flexibility to adapt and adjust the force output. Furthermore, Pethick et al.^11^ have suggested that during sustained or repeated contractions below the CT, the neuromuscular system maintains the required torque by recruiting additional motor units and/or increasing the firing rate of those motor units already recruited. Conversely, above the CT, it has been suggested that these adjustments are associated with a progressive loss of muscle metabolic homeostasis which indirectly affect the neural drive to the muscles and, therefore, the motor unit recruitment and firing rate, resulting in a loss in the neuromuscular system adaptability^11,19–23^. Interestingly, a recent work by Gomes and colleagues^24^ demonstrated that the neuromuscular fatigue-related changes in torque complexity are highly correlated with knee extensors’ motor unit behaviour. However, to the best of our knowledge, the contribute of motor unit behaviour to explain the relationship between force complexity and contraction intensity remains to be investigated.

This study aimed to investigate whether changes in motor unit recruitment and firing rate with contraction intensity would accompany and explain the inverted U-shape relationship between force complexity and contraction intensity. The literature suggests that the relative contribution of motor unit recruitment and firing rate strategies to the muscle force production varies depending on the muscle group and the force level. Specifically, motor unit recruitment is the primary mechanism used by the neuromuscular system at lower levels of force production, and the firing rate become the dominant mechanism at higher levels of force production, particularly beyond the upper limit of motor unit recruitment^25,26^. For the knee extensors, the literature suggests that, although the upper limit for this muscle group is typically at ~ 80–90% MVIC^27^, the motor unit recruitment increases steeply up to 40–50% MVIC, from which the rate of increase in motor unit recruitment becomes more gradual^28^. Thus, we hypothesised that both motor unit recruitment and firing rate would increase with contraction intensity until an intensity close to 40–50% MVIC; while beyond this intensity, we expect the motor unit recruitment to stabilize, and an increase based in firing rate adjustment. Furthermore, considering that force complexity provides information about the nonlinear interactions between multiple physiological mechanisms involved in the force production, we hypothesised that force complexity would peak at this range of force production, since it represents the range of force production in which both motor unit recruitment and firing rate would operate optimally, leading to a greater flexibility and ability of the motor system to adapt the force production to the force target, resulting in greater complexity. Moreover, we hypothesised that the changes in motor unit behaviour with the increase in contraction intensity could partly explain the inverted U-shape relationship between force complexity and contraction intensity.

## Materials & methods

### Participants

Twenty-five young and healthy male adults aged 18–35 years (age:22.8 ± 3.3; height:1.77 ± 0.06 m; body mass:70.5 ± 7.0 kg; body mass index:22.5 ± 1.6 kg/m^2^) were recruited and participated in the present study. Exclusion criteria included lower limb musculoskeletal injury within the last 6 months, or any neurological disorder. Prior to participation, written informed consent was obtained from all individual participants included in the study, previously approved by the Ethics Committee of the Faculty of Human Kinetics (approval number #3/2022). The procedures used in this study adhered to the tenets of the Declaration of Helsinki. Furthermore, participants were also instructed to avoid moderate to vigorous physical activity and resistance training during their participation in the study.

### Experimental design & protocol

The participants were asked to visit the laboratory on two different sessions. The first session involved the determination of the participants’ optimal knee extensors angle, their MVIC and their end-test torque (ETT)^29^. ETT was chosen in order to reduce the number of bouts of exhaustive exercise necessary to determine the CT. Moreover, Burnley^29^ has demonstrated that the ETT, obtained from a 5-min all-out protocol composed by 60 intermittent MVIC (3s contraction, 2 s rest), was not different from the CT determined using linear regression of the torque impulse and contraction time during five submaximal tests performed until the task failure.

**Fig. 1:**
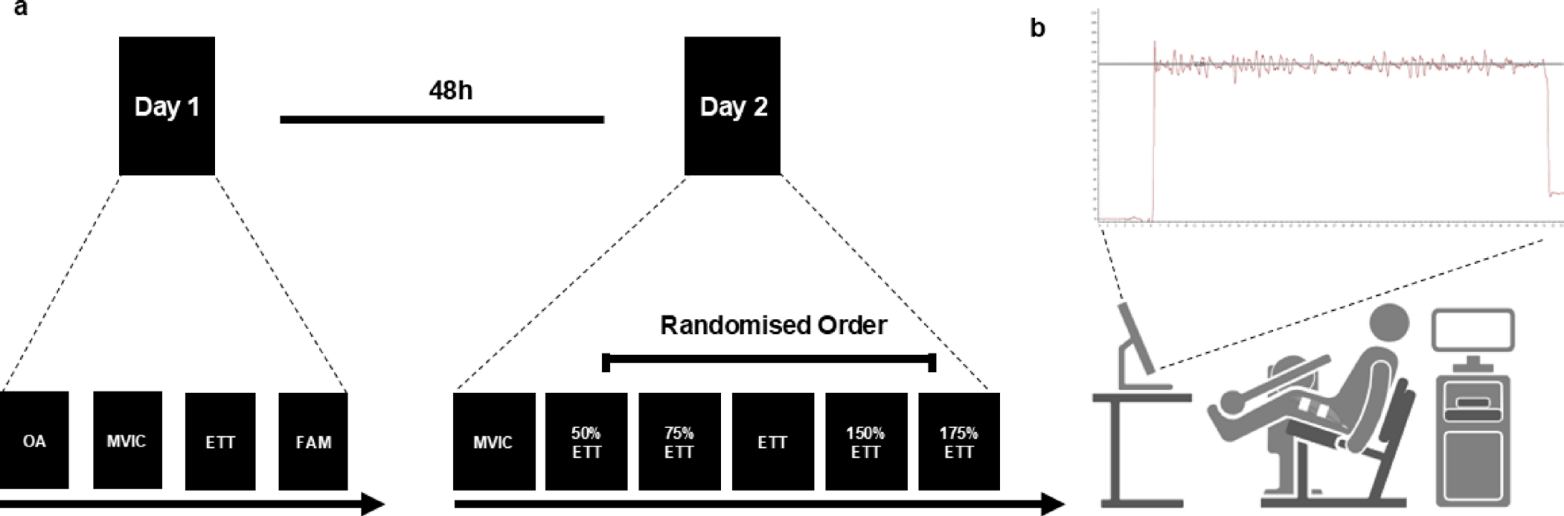
Schematic representation of **(a)** experimental design, and **(b)** experimental setup.

Furthermore, at the end of their first visit, participants were familiarised with all testing procedures that took place on the second visit. The second session corresponded to the experimental protocol in which participants were asked to perform a maximal task involving MVICs and a targeted submaximal sustained task through a continuum of five intensities corresponding to their ETT, 50%ETT, 75%ETT, 150%ETT and 175%ETT, as depicted in Fig. 1. These intensities were selected in order to be clearly outside of the confidence limits of 5–10% in which the critical torque has been suggested to be a phase transition instead of a threshold^21^ and to cover a broad spectrum of intensities. Both maximal and submaximal tasks were assessed at the participants’ optimal knee extensors angle. These two sessions were separated by at least 48 h and were conducted at the same time of the day to avoid daily variations of muscle force related to human circadian rhythms^30^.

### Data collection

All testing procedures were performed using an isokinetic dynamometer (Biodex Medical System Pro 3, Shirley, NY, USA), initialised and calibrated according to the manufacturer’s instructions. The isokinetic dynamometer’s seat was adjusted to guarantee the alignment between the lateral epicondyle of the femur and the axis of rotation of the lever arm, in which the participant’s dominant lower leg was attached above the malleoli with a velcro strap. The full extension was defined as 0º, and the chair was adjusted to ensure relative hip and knee angles at 90°. Straps were then firmly secured across both shoulders and waist to prevent extraneous movement and the use of the hip extensors during the contractions. To get participants familiarised with the testing tasks and to ensure a proper warm-up, participants performed a range of submaximal isometric, isokinetic and isotonic knee extensions before testing.

During the first visit to the laboratory, to determine the optimal knee extensors angle, participants were asked to complete five consecutive, maximal voluntary isokinetic concentric contractions at 60 deg.sec^−1^ through an 110º range of movement. The optimal angle was determined as the articular position corresponding to the best peak torque (PT) value, i.e. the highest torque value, obtained during the five isokinetic contractions. After a rest period of 10 min, participants performed a series of brief MVIC, lasting 3–5 s, to establish their isometric PT. These contractions were separated by 60 s of rest and continued until three consecutive peak torques were within 5% of each other. Participants were asked to employ their maximum force against the lever arm of isokinetic dynamometer set at 70º of flexion, as fast as possible, with strong verbal encouragement provided by the researcher. Then, after a proper rest period, the ETT was assessed as described in Burnley^29^. Briefly, the participants performed a 5-minutes all-out test composed by 60 intermittent MVIC (3s contraction, 2 s rest). Lastly, after a proper period of rest, the participants performed a series of sustained submaximal isotonic contraction to get familiarised with the testing procedures of the second session.

On the second visit to the laboratory, participants were asked to perform a series of brief MVIC to establish their daily PT as in the first session. For the submaximal trials, participants were asked to perform a hold isometric task consisting in sustaining a resistance equivalent to their ETT, 50%ETT, 75%ETT, 150%ETT and 175%ETT. A hold type of isometric contraction, that is a condition in which the lever arm of isokinetic dynamometer arm is not blocked and the participant is asked to hold a certain position at a given torque, was used given the recent observed differences as compared to push type of contraction^31^. Thus, the isotonic mode of the isokinetic dynamometer was used. To avoid unnecessary fatigue, the lever arm was placed at participants’ optimal knee extensors angle by the researcher. Then, whenever the researcher reached the optimal knee extensors angle, participants were instructed to maintain the joint angle position and to match their instantaneous torque with a target line superimposed on a display in front of them, equivalent to the resistance applied by the isokinetic dynamometer. The resistance equivalent to individual’s ETT was determined by multiplying the PT by the %MVIC equivalent to the ETT obtained during the first session. From this value, the intensity corresponding to 50%ETT, 75%ETT, 150%ETT and 175%ETT was calculated. For each intensity, two trials of 30 s, with a 1-minute resting period in between, were conducted. The 30-second analysis window began once participants had stabilized their torque production, following the release of the isokinetic dynamometer lever arm by the researcher. Moreover, whenever it was possible, the recording duration was extended for 35 to 40 s, which enabled us to select a 30-second segment that was as stable and representative as possible of the phenomena, for analysis. This approach allowed us to avoid both transient fluctuations associated with movement initiation, and fatigue related adjustments. Further, if the testing angle varied by more than 10º from the optimal angle, the trial was interrupted immediately and an additional trial was performed. The order of intensities was randomized.

Data were sampled through Biopac MP100 (Biopac Systems Inc., California, USA) interfaced with a personal computer. All signals were sampled at 1000 Hz. Data were collected through Acknowledge (Version 3.9.1. Biopac Systems, Inc., California, USA) and further exported to Matlab^®^ R2023a (The MathWorks Inc., Natick, MA, USA).

Regarding electromyography (EMG), surface EMG was acquired using two bipolar high-density surface electrodes (Trigno Galileo, Delsys, Natick, USA) placed on *vastus lateralis* (VL) and *vastus medialis* (VM) and placed according to SENIAM (Surface EMG for Non-Invasive Assessment of Muscles) recommendations, aligned with muscle fibers^32^. Prior to the electrodes’ placement, the skin was prepared by hair removal and cleaned with alcohol to improve signal quality. The electrodes were securely fixed with additional tape to prevent movement artifacts. EMG signals were preamplified and band-pass filtered between 20 and 450 Hz, while collected at 2000 Hz. EMG data were collected through EMG Works (Delsys, Natick, USA).

### Data analysis

All data from the experimental protocol were analysed using custom code developed in Matlab^®^ R2023a (The MathWorks, Natick, MA, USA). Torque signals were first low-pass filtered (Butterworth 10 Hz, 4th order), and then cropped to remove the ascending and descending components of the hold isometric contractions, as well as the first and last seconds of each trial. This procedure has guaranteed that the analysed signals only accounted for the time the participant matched the targeted torque level. Afterwards, a power spectral analysis was employed and revealed the 10 Hz to be the maximal frequency of interest across all participants. The signals were further downsampled to 50 Hz for analysis, following the best practices recommended by Stergiou^33^ of a sampling frequency five times greater than the highest frequency of interest in the time series. Then, the magnitude of force variability, through the coefficient of variation (CV), and temporal structure of force variability, through Sample Entropy (SampEn)^34^ were calculated. CV was calculated through the standard deviation normalized to the mean. SampEn, a measure of the regularity of the signal, was determined following the recommendations of Richman and Moorman^34^. In particular, SampEn determines the inverse probability that short sequences of data points are repeated throughout a temporal sequence of points. Therefore, a regular signal, characterised by perfectly repeatable time series has a SampEn value equal to zero, while and a random time series has a SampEn value converging towards infinity, typical of irregular signals^34^. In this study, for SampEn calculation, we used a pattern length (m) of 2, an error tolerance (r) of 0.2 and a data length (N) of 1500 data points (i.e., 50 Hz x 30 s)^35,36^. These inputs values, when applied to all trials and participants, have resulted in optimal reliability for entropy measures^37^. SampEn and CV were extracted from the exact same previously cropped signal. For statistical purposes, the average of the two trials in each intensity was used.

For EMG signals, data from VL and VM were decomposed using the Neuromap System (Delsys, Natick, USA) to extract the motor units action potential (MUAP) trains of concurrently active motor units^38^. The accuracy of each decomposed MUAP train was calculated as previously recommended^39^. For the present study, we have only analyzed MUAP trains that were decomposed with an accuracy level greater than 90%, as previously done by Contessa et al.^40^. For analysis, we obtained the average firing rate (FR_a_), calculated from the inverse of the inter-pulse intervals the constant-force segment of each submaximal contraction, and motor unit action potential amplitude (MUAP_a_), calculated as the maximum amplitude of the positive or negative MUAP phases detected across the four EMG channels^40^.

### Statistical analysis

All statistical analyses were conducted in Rstudio (version 4.3.1, R Core Team 2023). Standard descriptive statistics (mean and standard deviation) were used to summarise the data. All variables were tested for normality using Shapiro-Wilk tests. To investigate the effect of contraction intensity both on variability-related measures (SampEn and CV) and motor unit behaviour-related measures (FR_a_ and MUAP_a_), a Linear Mixed Model with Repeated Measures (LMM-RM) was performed^41^. Contraction intensity (50%ETT, 75%ETT, ETT, 150%ETT and 175%ETT) entered the model as fixed factor, and participants as random effect. For the FR_a_ and MUAP_a_ analysis, the number of decomposed motor units entered the model as covariate. This model was considered the most robust approach to analyse our data as it accounts for the individual variability, missing values and the potential effect of the number of decomposed motor units^41^. Tukey-adjusted 95% paired-samples confidence intervals (95% CI) were used to perform post-hoc analysis. Partial eta squared ($\:{\eta\:}_{p}^{2}$) and Cohen’s d were used to test the effect sizes for the LMM-RM and post-hoc analyses, respectively.

Additionally, a stepwise multiple linear regression analysis was conducted to predict the torque SampEn based on motor unit behavior related variables. Levels of *F* to enter and *F* to remove were set to correspond to *p* levels of 0.05 and 0.10, respectively. Statistical significance was set at *p* < 0.05.

## Results

### Torque’s sample entropy and coefficient of variation

All data regarding the temporal structure and magnitude of torque fluctuation are summarised in Table 1.

**Table 1:** Sample entropy (SampEn) and coefficient of variation (CV) during contractions at intensities equivalent to end-test torque (ETT), 50%, 75%, 150% and 175% ETT.

	Torque (*N*.m)	Torque (%MVIC)	SampEn		CV	
Condition			Mean ± SD	95% CI	Mean ± SD	95% CI
**50% ETT**	56.4 ± 13.3	17.9 ± 4.2	1.02 ± 0.21^a, b,c^	[0.93,1.10]	4.07 ± 0.63	[3.81,4.83]
**75% ETT**	84.6 ± 19.9	26.9 ± 6.3	1.11 ± 0.21^a, b^	[1.03,1.20]	4.18 ± 2.61	[3.11,5.26]
**ETT**	112.9 ± 26.5	35.8 ± 8.5	1.29 ± 0.17	[1.22,1.36]	3.90 ± 0.91	[3.51,4.29]
**150% ETT**	169.3 ± 39.8	53.7 ± 12.7	1.29 ± 0.25	[1.18,1.39]	3.80 ± 0.61	[3.54,4.07]
**175% ETT**	197.5 ± 46.4	62.7 ± 14.8	1.23 ± 0.31	[1.09,1.36]	4.18 ± 2.38	[3.17,5.19]

SampEn sample entropy, CV coefficient of variation, SD standard deviation, CI confidence interval.

Symbols indicate a statistically significant difference compared to the following ^a^ETT, ^b^150% ETT and ^c^175% ETT.

**Fig. 2:**
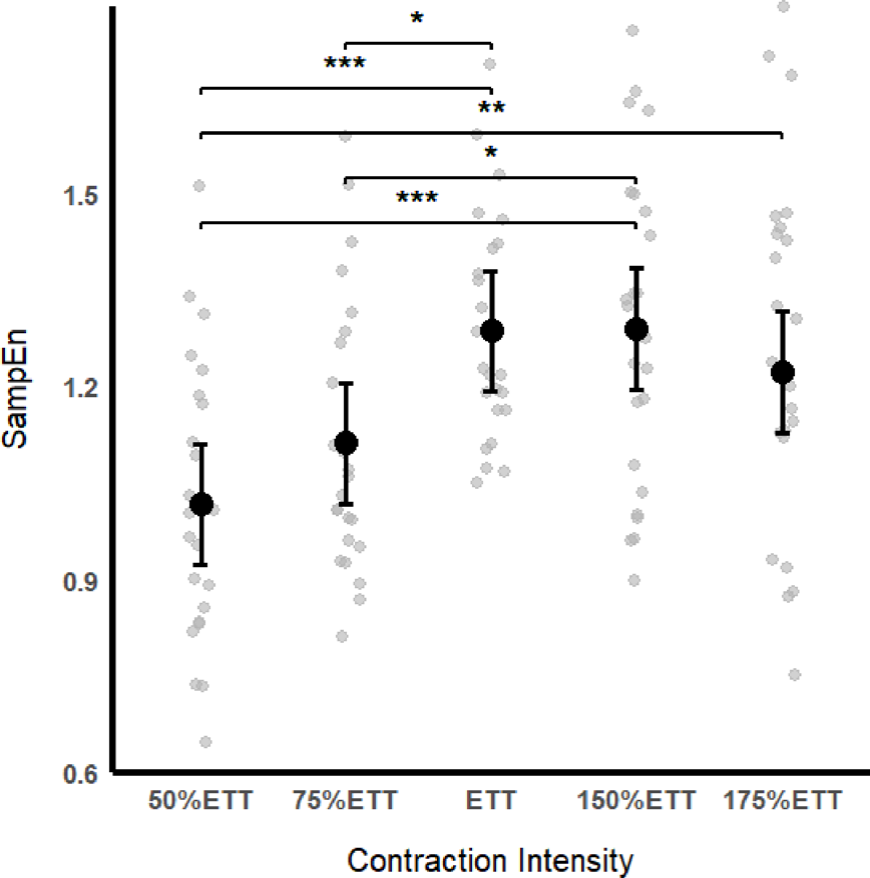
Relationship between Sample Entropy and contraction intensity. Average data are presented as Mean with 95% Confidence Interval in black circles. Grey circles represent individual data.

Regarding the temporal structure of torque fluctuations, we observed a significant fixed effect of contraction intensity in SampEn (F_(4, 94.4)_ = 9.55, *p* < 0.001, $\:{\eta\:}_{p}^{2}$= 0.290). As depicted in Fig. 2, post hoc comparisons showed SampEn at ETT to be significantly higher than at 50% ETT (MD = 0.27, t(94)=5, *p* < 0.001, d = 1.41) and at 75% ETT (MD = 0.17, t(94)=3.24, *p* = 0.014, d = 0.92). Also, SampEn at 150% ETT was significantly higher than at 50% ETT (MD = 0.27, t(94)=4.98, *p* < 0.001, d = 1.43) and at 75% ETT (MD = 0.18, t(94)=3.25, *p* = 0.014, d = 0.93). SampEn at 175% ETT was also higher than at 50% ETT (MD = 0.20, t(94) = 3.76, *p* = 0.003, d = 1.08).

For the magnitude of variability, no significant effect of contraction intensity was observed in CV (F_(4, 120)=_0.495, *p* = 0.739, $\:{\eta\:}_{p}^{2}$=0.02).

### Motor unit action potentials (MUAP) and firing rate (FR)

The data relative to the motor unit parameters are presented in Table 2.

**Table 2:** Decomposed EMG measures of Vastus lateralis and Vastus medialis during contractions at intensities equivalent to end-test torque (ETT), 50%, 75%, 150% and 175% ETT.

			MUAP_a_ (µV)		FR_a_ (pps)	
Condition	MU (Nr.)	Accuracy (%)	Mean ± SD	95% CI	Mean ± SD	95% CI
**Vastus Lateralis**						
**50% ETT**	15.5 ± 4.24	95.2 ± 0.90	0.059 ± 0.026^b, c,d^	[0.048,0.069]	6.94 ± 1.39^a, b,c, d^	[6.40,7.49]
**75% ETT**	16.9 ± 4.59	95.3 ± 0.96	0.095 ± 0.076^c, d^	[0.065,0.124]	7.81 ± 1.55^b, c,d^	[7.20,8.41]
**ETT**	16.3 ± 4.68	95.5 ± 0.97	0.125 ± 0.096 ^c, d^	[0.088,0.163]	8.69 ± 1.48^c, d^	[8.11,9.27]
**150% ETT**	17.1 ± 4.19	95.5 ± 0.61	0.193 ± 0.160	[0.130,0.256]	9.70 ± 1.29	[9.20,10.20]
**175% ETT**	16.9 ± 4.64	95.6 ± 0.76	0.200 ± 0.100	[0.161,0.239]	9.61 ± 1.21	[9.14,10.10]
**Vastus Medialis**						
**50% ETT**	15.9 ± 3.47	95.0 ± 1.01	0.058 ± 0.027^b, c,d^	[0.047,0.068]	7.20 ± 1.55^a, b,c, d^	[6.59,7.81]
**75% ETT**	17.4 ± 4.32	95.2 ± 0.91	0.083 ± 0.044^c, d^	[0.066,0.101]	7.84 ± 1.76 ^b, c,d^	[7.15,8.53]
**ETT**	20.9 ± 4.29	95.5 ± 0.87	0.114 ± 0.055 ^c, d^	[0.092,0.136]	8.54 ± 1.95^c, d^	[7.77,9.30]
**150% ETT**	18.3 ± 4.00	95.0 ± 1.22	0.180 ± 0.107	[0.138,0.222]	9.37 ± 1.86	[8.64,10.1]
**175% ETT**	19.3 ± 5.52	95.1 ± 0.82	0.215 ± 0.138	[0.161,0.269]	9.33 ± 1.73	[8.65,10.0]

MUAP_a_ motor unit action potentials amplitude, FR_a_ average firing rate, SD standard deviation, CI confidence interval. Symbols indicate a statistically significant difference compared to the following: ^a^ 75% ETT, ^b^ ETT, ^c^ 150% ETT, ^d^ 175% ETT.

For motor unit recruitment, we observed a significant fixed effect of contraction intensity in VL MUAP_a_ (F_(4, 95.3)_ = 25.8, *p* < 0.001, 

$\:{\eta\:}_{p}^{2}$= 0.51). No effect of the number of motor units was observed (F_(1, 108.9)_=0.00004, *p* = 1.0) Post hoc comparisons revealed MUAP_a_ at 50% ETT to be significantly lower than at ETT (MD=−0.07, t_(95.1)_=−3.86, *p* = 0.002, d=−1.09), at 150% ETT (MD=−0.13, t_(95.4)_=−7.69, *p* < 0.001, d=−2.2), and at 175% ETT (MD=−0.14, t_(95.3)_=−8.11, *p* < 0.001, d=−2.31), as at 75% ETT to be significantly lower than at 150% ETT (MD=−0.10, t_(95)_=−5.69, *p* < 0.001, d=−1.61) and at 175% ETT (MD=−0.11, t_(95)_=−6.09, *p* < 0.001, d=−1.72), and at ETT to be significantly lower than at 150% ETT (MD=−0.07, t_(95.1)_=−3.90, *p* = 0.002, d=−1.11) and at 175% ETT (MD=−0.07, t_(95.1)_=−4.31, *p* < 0.001, d=−1.22). For VM muscle, the results showed a significant effect of contraction intensity in MUAP_a_ (F_(4, 95.3)_=36.8, *p* < 0.001, $\:{\eta\:}_{p}^{2}$= 0.61). No effect of the number of motor units was observed (F_(1, 101.7)_ = 3.35, *p* = 0.07). Post hoc comparisons revealed significant differences between MUAP_a_ at 50% ETT and at ETT (MD=−0.04, t_(96)_=−2.82, *p* = 0.046, d=−0.87), at 150% ETT (MD=−0.12, t_(95.3)_=−7.85, *p* < 0.001, d=−2.26) and at 175% ETT (MD=−0.14, t_(95.5)_=−9.84, *p* < 0.001, d=−2.90), between MUAP_a_ at 75% ETT and at 150% ETT (MD=−0.09, t_(95)_=−6.48, *p* < 0.001, d=−1.84), and 175% ETT (MD=−0.13, t_(95.2)_=−8.62, *p* < 0.001, d=−2.47), and between MUAP_a_ at ETT and 150% ETT (MD=−0.07, t_(95.1)_=−4.82, *p* < 0.001, d=−1.40) and at 175% ETT (MD=−0.10, t_(95.1)_=−7.11, *p* < 0.001, d=−2.03).

**Fig. 3:**
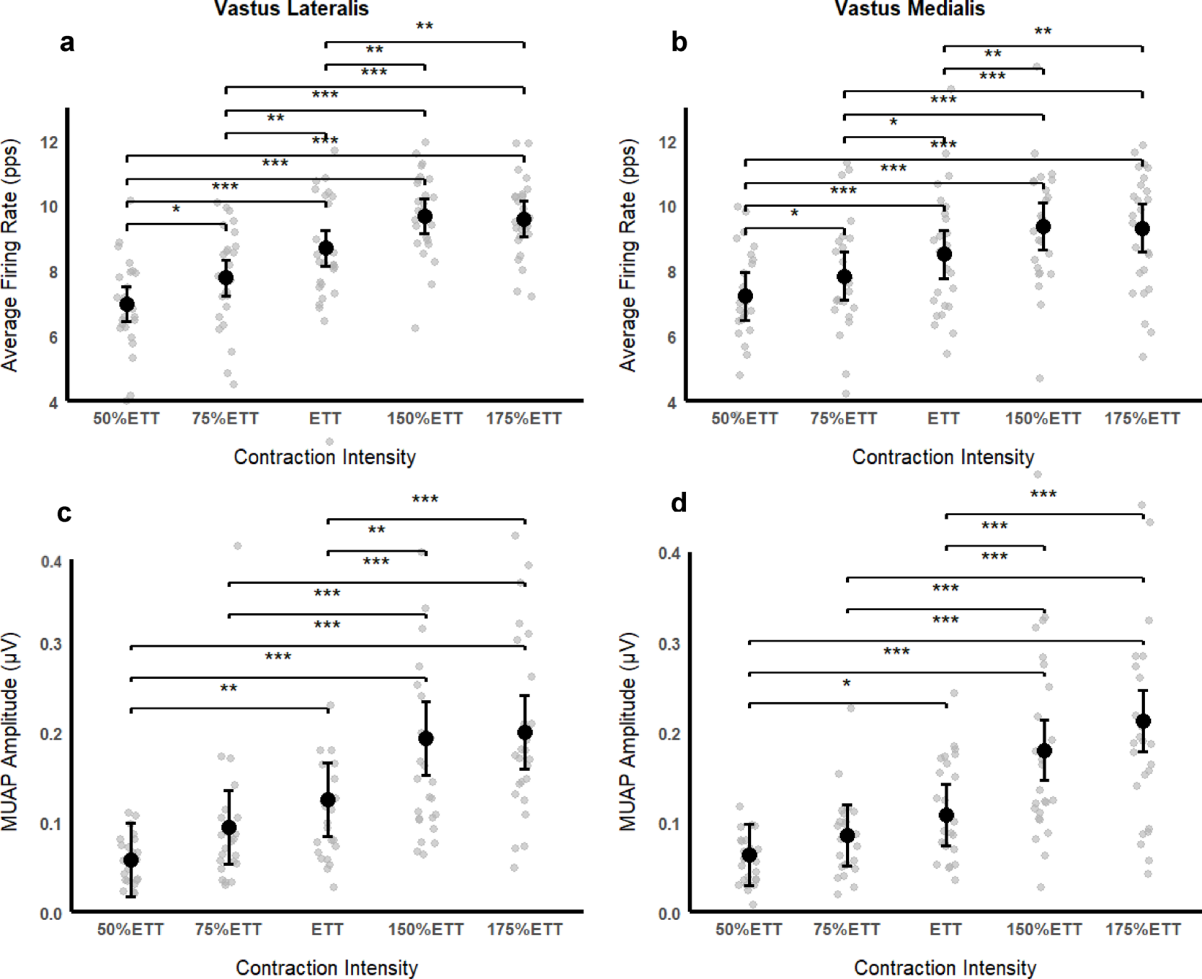
Motor unit behaviour across the different contraction intensities: **(a)** and **(c)** represent the behaviour of average firing rate and MUAP amplitude across different intensities for vastus lateralis, respectively. **(b)** and **(d)** represent the behaviour of average firing rate and MUAP amplitude across different intensities for vastus medialis, respectively. Average data are presented as Mean with 95% Confidence Interval in black circles. Grey circles represent individual data. MUAP stands for motor unit action potential (in micro volts); ETT refers to end-test torque; pps stands for pulses per second. *, ** and *** indicates *p* < 0.05, *p* < 0.01 and *p* < 0.001, respectively.

Regarding firing rate, there was a significant effect of contraction intensity in VL FR_a_ (F_(4, 95)_=39.6, *p*<0.001, $\:{\eta\:}_{p}^{2}$= 0.63). No effect of the number of motor units was observed (F_(1, 112.8)_=2.99, *p*=0.08). Post hoc comparisons showed VL FR_a_ at 50% ETT to be significantly lower than at 75% ETT (MD=−0.81, t_(95.4)_=−3.09, *p*=0.02, d=−0.88), at ETT (MD=−1.72, t_(95.1)_=−6.61, *p*<0.001, d=−1.88), at 150% ETT (MD=−2.69, t_(95.5)_=−10.3, *p*<0.001, d=−2.94) and at 175%ETT (MD=−2.61, t_(95.4)_=−9.98, *p*<0.001, d=−2.85); VL FR_a_ at 75% ETT to be significantly lower than at ETT (MD=−0.91, t_(95.1)_=−3.51, *p*=0.006, d=−0.99), at 150% ETT (MD=−1.89, t_(95)_=−7.28, *p*<0.001, d=−2.06) and at 175% ETT (MD=−1.80, t_(95)_=−6.95, *p*<0.001, d=−1.97); and VL FR_a_ at ETT to be significantly lower than at 150% ETT (MD=−0.98, t_(95.1)_=−3.43, *p*=0.003, d=−1.07) and at 175% ETT (MD=−0.89, t_(95.1)_=−3.43, *p*=0.008, d=−0.97). For VM muscle, we observed significant effect of contraction intensity in VM FR_a_ (F_(4, 95.1)_=36.3, *p*<0.001, $\:{\eta\:}_{p}^{2}$= 0.61). No effect of the number of motor units was observed (F_(1, 97.7)_=0.27, *p*=0.600). Post hoc comparisons demonstrated significant differences between VM FR_a_ at 50% ETT and at 75% ETT(MD=−0.62, t_(95)_=−2.94, *p*=0.033, d=−0.84), at ETT (MD=−1.29, t_(95.4)_=−5.63, *p*<0.001, d=−1.73), at 150% ETT (MD=−2.15, t_(95.1)_=−9.98, *p*<0.001, d=−2.88) and at 175% ETT (MD=−2.10, t_(95.2)_=−9.55, *p*<0.001, d=−2.81); between VM FR_a_ at 75% ETT and at ETT (MD=−0.67, t_(95.2)_=−3.03, *p*=0.026, d=−0.9), at 150% ETT (MD=−1.52, t_(95)_=−7.20, *p*<0.001, d=−2.04) and at 175% ETT (MD=−1.47, t_(95.1)_=−6.89, *p*<0.001, d=−1.98); and between VM FR_a_ at ETT and at 150% ETT (MD=−0.86, t_(95.1)_=−3.95, *p*=0.001, d=−1.15) and at 175% ETT (MD=−0.81, t_(95)_=−3.78, *p*=0.002, d=−1.08), as depicted in the figure 3.

### Multiple linear regression

Multiple linear regression was used to test if motor unit related measures (MUAP_a_ and FR_a_) from both the vastus lateralis and vastus medialis significantly predicted SampEn. The fitted regression model was:

$$\:SampEn=0.61-650.55*VL\:MUAPa+0.08*VL\:FRa,$$

where SampEn stands for Sample Entropy, and VL MUAP_a_ and VL FR_a_ stand for motor unit action potential and firing rate of vastus lateralis muscle, respectively. This model was chosen as it provided the best fit to the data based on Akaike Information Criterion (AIC) and Bayesian Information Criterion (BIC) values.

The overall regression was statistically significant (F_(2, 120)_ = 13.19, R^2^ = 0.18, *p* < 0.001, AIC = −5, BIC = 6.25), indicating that SampEn is higher when VL MUAP_a_ and VL FR_a_ present lower and higher values, respectively.

## Discussion

This study aimed to investigate whether changes in motor unit recruitment and firing rate with contraction intensity would accompany and explain the inverted U-shape curve that characterizes the relationship between force complexity and contraction intensity. We hypothesised that both motor unit recruitment and firing rate would increase with contraction intensity until an intensity correspondent to 40–50% MVIC, while beyond this intensity, we expected the motor unit recruitment to run out, and an increase in firing rate would occur. This hypothesis was partially supported by our results since we observed that both strategies increased with contraction intensity until 150% ETT (equivalent to ~ 52%MVC). As expected, at 175% ETT, we observed no further changes in MUAP, evidenced by no differences between the higher intensities’ conditions. However, contrary to our hypothesis, we also observed no further increases in firing rate at this intensity. Furthermore, we hypothesised that these changes in motor unit behaviour could explain the relationship between force complexity and contraction intensity. Interestingly, our findings demonstrated that MUAP and FR partly explain the changes in force complexity.

Force complexity has been suggested to be a key indicator of the neuromuscular system’s ability to adapt the force output to the task demands and/or environmental changes, i.e. its adaptability^5^. Indeed, to regulate the force production, the neuromuscular system relies on multiple mechanisms such as motor unit recruitment, motor unit firing rates, motor unit synchronization, feedback loops, and attentional control^8^whose interactions results in complex patterns in force outputs.

Motor unit recruitment and firing rate are known to be two main neural mechanisms involved in the muscle force regulation. Specifically, motor unit recruitment is the primary mechanism used by the neuromuscular system at lower levels of force production, allowing gradual increases in the intensity of muscle contraction through the activation of progressively larger motor units^26,39,42^. However, it is also the mechanism that first runs out. For the knee extensors, the capacity to recruit motor units goes up to 40–50%MVIC^28^. Further increases in force production are achieved through increases in the firing rate of the recruited motor units which becomes the main mechanism by which the neuromuscular system regulates the production of force for higher intensities, optimizing the mechanical response of each muscle fiber^43–47^.

Slifkin and Newell^18^ have demonstrated an inverted U-shape relationship between force complexity of the hand muscles and contracting intensity, with the highest values of force complexity being reached between 30 and 40% MVIC, the percentage previously reported at which the motor units of hand muscles are fully recruited^44^. Although the authors did not collect data from EMG activity, they hypothesized that in this range of force production, the neuromuscular system would be able to engage both neural mechanisms (motor unit recruitment and firing rate) leading to a greater flexibility and ability of the motor system to adapt the force production to the force target, resulting in greater complexity. The present study extended the work of Slifkin and Newell^18^ to the knee extensors, demonstrating a trend to an inverted U-shape relationship between force complexity and contraction intensity, reaching the highest values of SampEn between ~ 35% and ~ 52% MVIC. Importantly, the present study extended these authors’ work by assessing the motor unit behaviour. Overall, we found the motor unit recruitment, measured through MUAP, and the firing rate of VL and VM muscles to increase with the contraction intensity until the 150% ETT condition (Table 2), an intensity equivalent to ~ 52% MVIC (Table 1). From the 150% ETT to 175% ETT, we observed no significant differences in the motor unit recruitment nor for the firing rate of the recruited motor units of both VL and VM. Regarding motor unit recruitment, since the contraction intensities used in ETT, 150%ETT and 175%ETT were equivalent to ~ 35%, ~ 53% and ~ 62% MVIC, respectively, our results align with the literature that suggest the 40–50% MVIC as the range of the maximal capacity of motor unit recruitment of the knee extensors^28^. On the other hand, contrary to what we expected, we observed no differences in motor unit recruitment between ‘neighbour’ intensities, despite changes in the firing rate. For instance, we observed no significant differences between 50% ETT and 75%ETT conditions, nor between 75% ETT and ETT conditions and ETT and 150% ETT (Fig. 2). This could be related to the well-known ‘onion-skin’ scheme of motor unit firing rates in which earlier-recruited motor units maintain higher firing rates than later-recruited ones at any time and contraction force level^39,48^. Based on this rationale, the increase in the contraction intensity could be achieved through the increase in the firing rate of recruited motor units with no need of additional motor units to be recruited. Furthermore, considering the 40%−50% MVIC as the range of maximal motor unit recruitment capacity, it would be plausible to expect an increase in firing rate in higher intensities. However, we observed no significant differences in firing rate between 150%ETT and 175% ETT which could be explained by the fatigue caused by sustaining the muscle force production at higher intensities. Interestingly, several studies have demonstrated that the normal voluntary control of motor units changes with fatigue, reporting the firing rates of active motor units to decrease while the excitation to the motoneuron pool increases to recruit additional motor units and increase their firing rates^48–50^. Moreover, it is known that above the ETT, peripheral fatigue develops ~ 4–5 times faster^19^ which could explain the firing rate results observed in 175% ETT condition. However, no direct or indirect (e.g. subjective measures such as rate of perceived effort) measures of fatigue were collected during this experiment, which represents a limitation that prevented us from testing this hypothesis.

Altogether, the changes observed in motor unit behaviour could explain the results obtained regarding force complexity. Interestingly, we observed that similar to MUAP and FR, the SampEn increased with the contraction intensity until the range of intensities between ETT and 150% ETT with a slightly, but not significant, decrease at 175% ETT. Thus, as suggested by Slifkin and Newell^18^ the range of intensities between ETT and 150% ETT could represent the range of force output in which both force regulation strategies - motor unit recruitment and firing rate – would optimally operate together, increasing the number of solutions available to the neuromuscular system to adapt the force output to meet the force target. Thus, this broader range of solutions leads the neuromuscular system to be more flexible and adaptable, resulting in increased force complexity values. Our results support this hypothesis since we observed MUAP to stabilize and FR to be maximal at 150% ETT. Conversely, as the force requirement increased in 175% ETT, we observed a slightly decrease in firing rate, reducing the number of solutions available for the neuromuscular system to adapt the force output, reflecting a decreased ability of the neuromuscular system to explore and achieve control solutions which lead to a decrease in force complexity^5^.

Considering that force complexity has been suggested to provide information about the multiple mechanisms involved in the production of complex force outputs^5,8–10^ in which the motor unit recruitment and firing rate are included, it would be expected that these two neural mechanisms could partly explain the changes in force complexity with the increase in contraction intensity. Interestingly we observed that force complexity was explained in 18% by the *vastus lateralis*’ motor unit behaviour. This result is in line with recent literature suggesting that force complexity changes are highly correlated with the motor unit behaviour of the knee extensors^24^. In particular, Gomes and colleagues^24^ demonstrated that the force complexity response to neuromuscular fatigue is correlated with the individual’s motor unit behaviour profile pre-fatigue, highlighting the association between motor unit parameters and force complexity changes with fatigue. Our findings extends those previous results by focusing on contraction intensity rather than fatigue, and by exploring the association between the changes in motor unit behaviour and force complexity across a range of intensities anchored to individual’s ETT, thus providing a novel perspective on the relationship between neuromuscular output and force complexity.

Further, the linear regression model revealed a negative relationship between MUAP amplitude and force complexity. While at first this may appear counterintuitive, considering that previous research has shown that motor unit recruitment increases with contraction intensity, this finding may reflect underlying physiological mechanisms. As contraction intensity increases, the neuromuscular system gradually recruits larger motor units with higher MUAP amplitudes. Although recruitment continues, the neuromuscular system may become less flexible at higher intensities due to a reduced pool of motor units available for being recruited and an increased reliance on larger, less adaptable units. This reduced adaptability could explain the observed decline in complexity. It is important to note that motor unit recruitment and firing rate modulation operate concurrently throughout all intensity levels, and the negative association between MUAP amplitude and torque complexity may reflect a shift in their relative contributions across the force continuum, reflecting a transition toward a more constrained neuromuscular strategy as force requirements increase. While a linear model provides a simplified view of this relationship, future studies may benefit from nonlinear or segmented modeling approaches to better capture this relationship between motor unit behaviour and torque.

Moreover, albeit the low explanatory power (18%), which physiological significance can be debated, it is important to note that the firing rate and the motor unit recruitment are only two of several mechanisms involved in the regulation of torque production, which may influence torque complexity values. Indeed, torque complexity represents the the interaction between multiple mechanisms that contribute to produce complex force outputs. Therefore it is plausible that other mechanisms such as motor unit synchronization, feedback loops from muscle afferents, muscle metabolic rate, attentional control and, most importantly, the complex interaction between these mechanisms could also affect the regulation of torque production and, consequently, force complexity, which strengthen the meaning of our finding. Furthermore, it is, important to highlight that these results should be interpreted with caution since in this study we collected EMG from the VL and VM, which represent a fraction of the several muscles contributing to knee extension torque. Moreover, the literature has been suggesting that force complexity may not fully originate from the nervous system, i.e. the information contained in the force output does not exclusively reflect the information sent by the nervous system, but appears to be affected by other physiological and structural factors^51^. Therefore, further studies should include the assessment of the EMG activity of the other knee extensors muscles, structural-related measures and markers of fatigue to better understand the mechanisms underlying force complexity’s changes. Regardless, our findings unravel new insights to force complexity control mechanisms under different contraction intensities.

We observed SampEn to change only above the ETT. ETT, the analogous form of critical power^52,53^ is thought to be a critical threshold for neuromuscular fatigue development^19^. Specifically, it has been suggested that below the CT/ETT, the changes in muscle metabolic environment, in terms of phosphorylcreatine, inorganic phosphate and pH, are too small to affect the neuromuscular system’s submaximal output to any significant degree^54^. This results in a capacity to maintain flexible motor control solutions and, hence, in a greater adaptability to changes in task demands^11^. Conversely, above the CT/ETT, the muscle metabolic homeostasis cannot be achieved, resulting in the development of peripheral fatigue that leads to a progressive reduction in the force-generating capacity^19^. Considering that, for knee extensors, the CT typically occurs at 25–35% MVIC^29^ (~ 36%MVIC in the present study) and the motor units are recruited up to 40–50% MVIC^28^ it would be expected a range of intensities above de CT in which the neuromuscular system would be able to adapt its output despite the accumulation of fatigue. Therefore, for higher intensities and considering the effect of fatigue in firing rate described above, the mechanisms of force control would be restricted, resulting in a less adaptable system and, thus, in a progressive loss of force complexity^11,19,21^. Our results align with the presented literature since we observed a trend for an inverted U-shape relationship between force complexity and contraction intensity, with the highest SampEn values being reached between ETT and 150%ETT (equivalent to ~ 36–54% MVIC).

The absence of a perfect U-shape relationship between force complexity and contraction intensity could be explained by the intensities used in present study that have ranged from ~ 17% to ~ 61%MVIC (Table 1). Since 61%MVIC is close to the intensity at which both neural mechanisms are operating optimally, it would be expected that if we used higher intensities, a perfect inverted U-shape relationship would be observed as in Slifkin and Newell^18^. The results of Pethick et al.^21^ supports this since they found progressively lower force complexity values as the contraction intensity increased up to values close to 200% CT. However, we have chosen not to test higher intensities due to the methodological challenges of sustaining the necessary force levels through the entire duration of the testing trials for reliable entropy calculations in force signals^35^. Interestingly, Fiogbe et al.^13^ have reported the same issue in their study of knee extensors in which they used a range of intensities up to 40%MVIC. Considering the observed inter-individual variability in terms of ETT, with values ranging from ~ 21–57%MVIC and a standard deviation of ~ 8%MVIC, this study also highlights the importance of assessing the individual ETT/CT instead of using the MVIC as a reference.

Interestingly, we observed no changes in the magnitude of variability, as measured through CV, across the different contraction intensities. This finding is consistent with the literature demonstrating that CV tend to decline from a high value at low contraction intensities to relatively constant values at moderate-to-high contraction intensities^55–57^such as those used in the present study. Moreover, our findings provide further support to previous research suggesting that magnitude- and complexity-based measures capture distinct aspects of motor output variability^9,58^. Indeed, in our study, while CV remained relatively constant, SampEn exhibited significant changes across the different intensities, suggesting that complexity-based measures are more sensitive to detect subtle changes in motor output’s dynamics that magnitude-based measures are insensitive to, as suggested by the literature^58^. Our findings reinforce the importance of including complexity-based analysis to a better understanding of motor control and its underlying mechanisms.

Furthermore, this experiment has several limitations worth noting. Most notably, the absence of a ramp contraction^59^ prevented participants from gradually increase their torque production to match the targeted torque level. Instead, due to the isotonic nature of the task, which is closer to the real-life environment, and to avoid unnecessary fatigue, the lever arm of the isokinetic dynamometer was placed on optimal angle and, then, the participants were asked to instantaneously match the targeted force level. This approach limited the direct identification of each motor unit recruitment and derecruitment thresholds as recommended by the literature^60^. Instead, this threshold was indirectly inferred from the motor unit action potentials’ amplitude, assuming the size principle. It represents a methodological limitation since the MUAP amplitude is influenced by the variability of motor unit size and their spatial distribution^60^. Moreover, the inclusion of a ramp-up phase could have enabled smoother tracking of motor unit recruitment and firing rates by the decomposition algorithm, while reducing potential signal artifacts or nonlinear transients in the EMG data caused by abrupt torque changes. Furthermore, the inclusion of only young male adults is a limitation of the present study. Considering the emerging evidence of sex-based differences in force control^61–63^ as well as the impact of the aging process on the loss of complexity^8,13^ future studies should explore whether the relationship between torque complexity and motor unit behaviour across different contraction intensities differs between sexes or changes across the lifespan.

The present findings advanced our understanding of the physiological mechanisms that explain the relationship between force complexity and contraction intensity, contributing for an adequate interpretation of the changes in force motor control. Specifically, we demonstrated a trend for an inverted U-shaped relationship between force complexity and contraction intensity, reaching peak between ETT and 150%ETT. Similarly, an increase in motor unit actions potentials and firing rate was observed as the intensity increased up to 150% ETT. Interestingly we observed that force complexity was explained in 18% by the *vastus lateralis*’ motor unit behaviour. The results suggest that changes in force complexity depend on contraction intensity and can be partly explained by alterations in motor unit behaviour that occur as contraction intensity increases, which could influence the capacity of the neuromuscular system to be flexible and to adapt the motor output to the changes in task demands.

## Data Availability

The datasets generated during and/or analysed during the current study are available from the corresponding author on reasonable request.
